# The new alchemy: Online networking, data sharing and research activity distribution tools for scientists

**DOI:** 10.12688/f1000research.12185.1

**Published:** 2017-08-03

**Authors:** Antony J. Williams, Lou Peck, Sean Ekins

**Affiliations:** 1 National Center for Computational Toxicology, Environmental Protection Agency, Durham, NC, 27711, USA; 2 Lou Peck Consulting, Swansea, SA4 3JQ, UK; 3 Collaborations Pharmaceuticals, Inc., Raleigh, NC, 27606, USA

**Keywords:** Online networking, Social networking, Research data sharing, altmetrics, Alternative metrics

## Abstract

There is an abundance of free online tools accessible to scientists and others that can be used for online networking, data sharing and measuring research impact. Despite this, few scientists know how these tools can be used or fail to take advantage of using them as an integrated pipeline to raise awareness of their research outputs. In this article, the authors describe their experiences with these tools and how they can make best use of them to make their scientific research generally more accessible, extending its reach beyond their own direct networks, and communicating their ideas to new audiences. These efforts have the potential to drive science by sparking new collaborations and interdisciplinary research projects that may lead to future publications, funding and commercial opportunities. The intent of this article is to: describe some of these freely accessible networking tools and affiliated products; demonstrate from our own experiences how they can be utilized effectively; and, inspire their adoption by new users for the benefit of science.

## Introduction

In the past 40 years, society has undergone considerable changes driven by the development of affordable personal computers, the internet and most recently, mobile devices, which allow widespread connection to the internet. In turn, these technologies have shaped how we interact with each other and form online networks. Since 2000 there has been an almost 800% increase in the number of people using the internet, with over 3.5 billion people online. Online sharing now ranges from posting opinions, 140 character nuggets on Twitter, updates and discussions on LinkedIn, photos or videos via free platforms such as YouTube or Vimeo, presentation slides on SlideShare and so on. The specific online media sites preferred in each country across the world differs. Despite the prevalence of social media tools, the vast majority of scientists do not use these tools to help share, evidence and amplify their *scientific research* ^1^. We believe there are a number of reasons that most scientists do not use these tools. It could be because few people would even think of using online media tools for their scientific research, or because they do not understand the potential value. The lack of credit for sharing pre-published data, code or other forms of research outputs, especially in terms of citations that can contribute to career progression, may also be an issue. Maybe scientists do not see this activity as a valuable use of their time or they require initial guidance to help navigate use of these tools. Other issues include fear of being scooped, and the idea that if your work is solid, it should be able to stand on its own merits without needing to be “amplified.”

 With new tools being developed, it is difficult to keep track, determine what works, and optimize their use, especially in the context of science. For example, Kramer and Bosman (2017) manage a growing list containing over 400 tools and innovations in scholarly communications, and some of these can be used for online sharing. This list reinforces the breadth of software tools available, and demonstrates how this can be confusing for any scientist to know where to start.

As the world has changed in the past three decades, scientific publishing, seen increasingly as the currency of science for at least 50 years, has also seen a dramatic shift. Since publishing of the first modern journal in 1665 (Le Journal des Sçavans), a staggering number of scholarly articles have been published, reaching the milestone of 50 million by 2009 ^2^. Recently Ware and Mabe ^3^ projected between 1.8–1.9 million scientific articles are now being published every year, while contributing one aspect of Big Data for science ^4^. In recent years, there has also been an explosion in the number of new Open Access publishers, a number of which seem to be focused on the profit potential of this new marketplace as predatory open access publishers. This exploitative open-access publishing business model charges publication fees without providing the editorial and publishing services associated with legitimate journals. This could allow scientists to game the system by publishing even poor quality science in predatory journals, effectively padding their resume. This was tested in an elaborate sting when a spoof research article was accepted by dozens of open access publishers ^5^. It even extended to allowing fake editors roles on their editorial boards ^6^. Such dubious practices in the publishing world, especially in terms of gaming a publication record can unfortunately extend to the use of online networking and sharing tools to boost online profiles and game altmetric scores ( *vide infra*).

**Figure 1.**
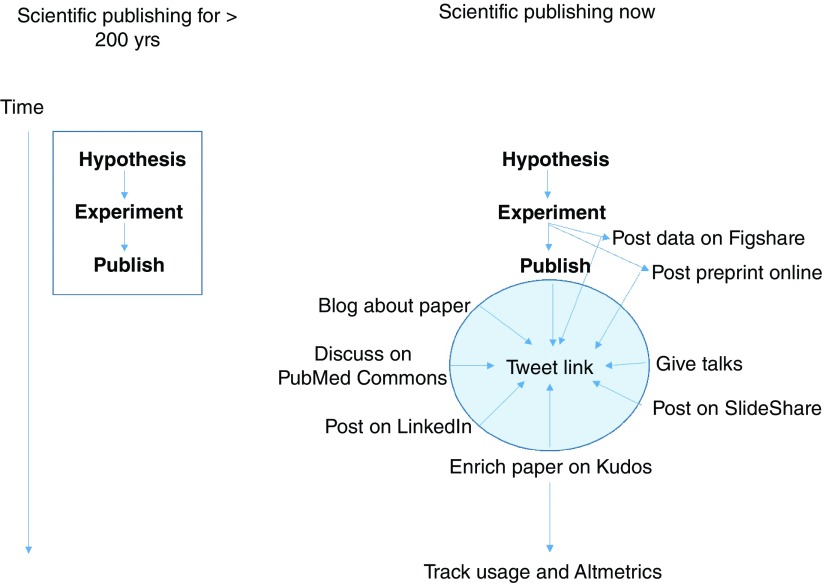
A comparison of science publishing over 200 years, then and now.

With such a large quantity of scientific research finding its way into published works, it is hard for scientists of any description, whether chemists, biologists or physicists to make sure their work rises above the ‘noise’. This is of course important if it is to be seen by peers and in turn used by them and cited, to ultimately be captured and further used to infer the importance for any given publication through various citation metrics. Historically, publishing was seen as the end result of the scientific endeavor, which one might consider as a simplistic linear process from hypothesis – experiment – publication ( Figure 1). However, the traditional approach of a particular piece of research dissemination reaching its conclusion once it has been published was a limitation of printed journals and should not pose such limitations in an electronic online world. Now, with the advent of internet-based technologies, the last decade has seen an explosive growth in additional forms of media for the dissemination of publications and associated research data, analyses and results, including wikis, blogs and code-sharing platforms (e.g. GitHub). The process of research publication is now non-linear, with a potentially infinite variety of dissemination steps that could be taken between hypothesis to publication and beyond ( Figure 1). This could include early release of data and scientific manuscripts as preprints. Sharing details regarding a research study and associated data offers a number of potential positive effects that can contribute to the quality of science. The historical approach of peer review was to hopefully both improve and ensure the quality of the science and the published output. Sharing research data and preprints allows for early feedback on both the results and preliminary findings from peer groups and reviewers.

This has many potential implications, one being a way to extend the life of a scientific publication and its enmeshing in a network of other electronic outputs. Another is the considerable effort that would be required to fulfill even a sampling of these approaches. Examples of the increasing importance of these non-traditional sources of scientific information is the increasing prevalence of links to Wikipedia, blog posts and code-sharing in reference lists associated with references in scientific publications ( ~35,000 citations to Wikipedia, ~11,000 for YouTube, ~10,500 for Facebook, and ~7000 for Twitter). This will increase in the future.

The vast majority of researchers depend on research funding in order to progress their science. However, acquiring research funding continues to be a challenge, with only 20.7% (14,457/69,973) of NIH Research project grants funded in 2015. This increased competition drives requirements to assess the quality and volume of research output. These exercises are becoming more commonplace, for example the Research Excellence Framework (REF2020) is the current system for assessing the quality of research in UK higher education institutions and, similarly, the Excellence in Research in Australia project (ERA). While there are various subjective judgements regarding the “impact” of a publication (including impact factors, CiteScore ^7^, h-index and citations) for a research effort and ultimately for the performance and impact of a scientist, it should be obvious that in our present time the sharing, networking and outreach of research work could bring benefits. What those benefits actually are, and how to deliver them reproducibly, is one of the mysteries of this new scientific networking ecosystem. How does one use technology to maximally increase the visibility of your research outputs and potentially lead to breakthroughs that benefit humanity? This could be seen as the ‘new alchemy’ of science. Suggested benefits might be broader reach for a publication and Altmetric scores ( *vide infra*) that could be used by granting agencies or others for assessing the scientist. By engaging or participating in online media for example, scientists can be empowered by using and adopting the tools available to them to share and improve access to their research, facilitating networking and potentially resulting in greater impacts on their career. However, these benefits come at a cost as they require time and effort that could distract from other efforts, such as research, writing grants, reviewing, mentoring and so on, and they are not yet in the standard workflow of the scientist.

Based on our collective experiences over the past five years, we believe there is a significant return on investment, and it is far higher than simply relying on publishers to invest informed and targeted efforts in sharing a scientific publication. This in fact rarely happens unless the publisher, or you, invest in a press release, but the vast majority of publications are certainly never picked up by the general press, primarily due to the sheer volume of scientific papers being published. For a publisher producing many thousands, if not tens of thousands of publications in a year, investing efforts to share information about one publication above and beyond one tweet, or a rudimentary blog post on their website, is highly unlikely. If publishers were to promote each article, book or report they publish, this would flood their communication channels, thus creating too much noise. Their own marketing efforts would be less effective through mass communication and may also reduce reach, especially to the overwhelmed specific target audiences that would benefit from the author's research. Ultimately, the best person to raise awareness is the author(s) themselves who can leverage their own networks, whether through electronic tools or personal interactions. These may be as effective as a press release when it comes to spreading the word on what is likely a hot topic relevant to few scientists in a specialized field.

A scientific publication is considered the most basic and historical path to “sharing” the product/s of one’s research. This is also generally considered the final output detailing the purpose for a particular piece of research, provides enough detail to reproduce the work and access to sufficient data (either directly in the manuscript, as supplementary info files, or via links to data stored on other external websites). Nowadays however, even a tweet with links to further scientific information or data can potentially represent a “ nanopublication”, although the longevity of some of these tools is questionable. This effort does not have to wait until publication as you could tweet ideas at the very earliest stages of the research depending on your level of openness, especially in regards to open data, as well as concern for being scooped by people following you. In the process of our own experiments to determine the benefits and paths towards online sharing of our research, we have identified at least four steps that the reader could put into action. These four steps are to explain, share, and gather evidence of increased awareness of the work, as well as gather feedback from the community. These efforts may only take a few minutes of a scientist’s time, which is a fractional investment compared with the hundreds to thousands of hours spent on the scientific research from conception to publication. These steps, in turn, may increase impact of the research efforts.

Investing additional efforts into sharing data, research outputs such as presentations, or the final published products of the research work, may directly benefit a scientist’s career (especially with the growing attention given to alternative metrics “ altmetrics”, *vide infra*), leading to new collaborations, new funding or even facilitate new discoveries. While there is certainly no shortage of software tools available to share and amplify research efforts, we will discuss a small number of free tools that we use ourselves, of course others are likely to have their own personal favorites. We hope this serves to whet the appetite, encourage further exploration to find out what tools work best for you to meet your objectives, and even form the foundation to discover new or other tools not identified in this piece.

## What are your goals at the start?

To extract the most value from these efforts it is certainly important to identify your primary goals and objectives for using these networking, sharing and amplification tools. For example, you might be interested in: 1) saving time by using integrated tools; 2) sharing your work using a small number of online services for dissemination and amplification; 3) tracking and providing evidence of your “impact” on research to potentially help with research funding applications; and/or 4) furthering your outreach to those outside your own network and perhaps engaging with the general public. Once you have defined your focus, you will be better positioned in terms of deciding what software services to use and whether to utilize a mixture or concentrate your efforts on one or two only. Your institution, colleagues, collaborators or even publishers may also have some recommendations for you or may already be partnered with specific services of which you can take advantage.

## Categories of tools: Networking, sharing, tracking and research amplification

We separated these software tools into four specific categories: networking, sharing, tracking and amplification. We acknowledge that the lines between many of these are actually rather fuzzy and that networking sites, such as Facebook and Twitter, are primarily used for sharing. Many of these tools actually serve more than one particular function, and our article is acknowledged to be subjective and based on our own experience and usage. There are obviously more generic tools that have long existed, for example email, and newer tools like Slack or Yammer, which are generally used for private communication and sharing. While these newer tools may be important for science and collaboration within small groups, they are useful in extending your online network more broadly. Clearly, the changes we have seen with technology may not predict what we will see in future in terms of communication style and utility as technologies themselves can redefine communication styles, as is the case with the 140 character tweet. Throughout this article we will point to examples of our own profiles on these various online tools as examples.

### Networking

**Figure 2.**
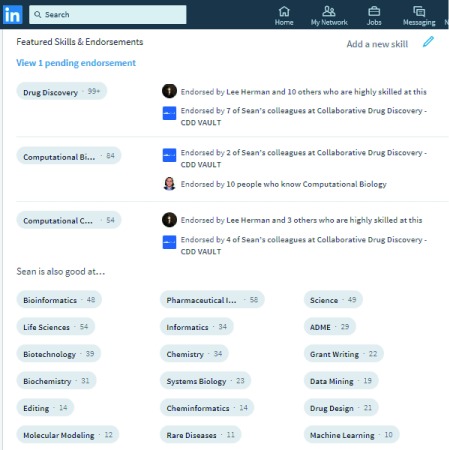
Endorsements for Sean Ekins on his LinkedIn profile. Note that these can be directly managed by the account owner to remove endorsements that they do not want to be visible.

***LinkedIn*.** While LinkedIn has a primary role to form connections and expose your career to potential employers and partners, it is likely the *de facto* networking tool for many professional scientists. LinkedIn is also the number one networking site for business and crosses all domains. The networking of scientists, business and investors offers potential opportunities for innovation and commercialization. Other platforms (e.g., ResearchGate) are more geared towards research. LinkedIn offers both free and paid levels of service and, to date, we have only used the free services. We think that anyone creating a LinkedIn profile should invest a minimal effort in terms of adding a “professional” photograph, a career history back to their research training (i.e. college or university), a minimum of two to three research publications, and links to public presentations on other sites ( *vide infra*). We also suggest that career interests be listed and some efforts invested in building out a network of work colleagues, ex-supervisors, co-authors, etc. We believe that LinkedIn be considered as an opportunity for career-networking, **not** family event and activity sharing, and to maintain the highest level of professionalism on this site. The ‘networking effect’ will likely result fairly quickly in new contacts reaching out to join your network, and we suggest being somewhat selective in accepting these offers. Some proposed filters for accepting new contacts include: 1) whether you have met them face-to-face; 2) the size of overlapping networks (for example when you have ~10 contacts in common); and 3) when you have already had either phone or email contact with the person. As well as recommendations from those you have worked directly with, the endorsement facility of LinkedIn allows members of your network to identify and endorse you for specific skills and this, in particular, can be valuable in terms of having a publicly acknowledged list of capabilities visible to the LinkedIn user base. An example of such an endorsement list is shown in Figure 2, which shows a partial list of the featured skills and endorsements for one of the authors (SE). This list can be pruned according to what skills you would want to be identified as having. It is also possible to directly list projects that you have been (or are) involved with, as well as associating the various members of the project team. This further defines the networking aspect of the site.

For those who use the site, they will have noticed business managers sharing their greatest coaching ideas or meaningful quotes. A scientist can use the same facility to post an update regarding present activities or simply something of scientific interest to share with followers. In this regard, the site can be considered as a more expansive version of Twitter. Some of these types of updates can be incredibly useful as tools to highlight a recent achievement for your group or collaborators. From our experience, a tendency towards keeping them brief and adding an image certainly helps in obtaining more views and ‘likes’. We have used this facility to provide updates on new papers or grants received as well as updates on specific projects. We have found that positive or engaging news can quickly gather momentum (e.g. announcing new grants, new hires, new jobs) and can drive contacts that lead to offline follow up. As an example, AJW posts updates on LinkedIn regarding his most recent project, the CompTox Chemistry Dashboard ^8^. The associated Google Analytics for the site shows that these postings are very effective in driving traffic to the site, commonly a few dozen visits within a day of posting an update about the site. This can be informative about your network and perhaps beyond, in that good news travels fast. One or more scientific publications can be associated with the profile to illustrate latest research efforts and we suggest association of your highest profile publications with the site. Ideally LinkedIn would make use of Digital Object identifiers ( DOIs) and would allow a resolver service, such as CrossRef, to display the publication details rather than forcing manual entry as it does at present. DOIs are one of the primary ways that scientific online tools integrate their data streams (i.e. ORCID, Altmetric, Publons). Besides using LinkedIn to share links to your latest publications, one can also insert PowerPoint presentations, PDF files and other document forms using the embed functionality available via SlideShare (described in more detail later).

The regularity of updates regarding new publications and presentations can be representative of your productivity, and requires active attention to your LinkedIn profile. The sharing of articles, presentations and science news that interests you can also help drive attention to other people’s work and elevate interest in it. For sharing via the site, we suggest using URL shortener services like TinyURL, Goo.gl URLs or Bitly to track the number of times people click on your link (as clickthroughs). However, it should be remembered that some services are currently banned in countries like China (e.g. Google), so you may want to ensure you are using “global friendly” services for this purpose. In our opinion LinkedIn is a pivotal tool and the purchase of LinkedIn by Microsoft ^9^ would indicate that it will be more closely integrated with their products, maybe even extending to include an integration to their Microsoft Academic site that operates in the same space as Google Scholar Citations. 

***ResearchGate and Academia*.** There are several tools for networking your publications. Two of the most popular are ResearchGate and Academia. While there are differences in functionality, both sites provide similar abilities in terms of sharing preprint manuscripts, presentations, posters and other forms of general communication of your science. For example, one author’s (AJW) Academia site lists ~250 publications, presentations, book chapters, magazine articles and other research outputs and has had ~30,000 views. The detailed analytics page includes geographical details, views and downloads and Academia’s measures of impact). A recent study in PLOS ONE ^10^ states that papers uploaded to Academia receive a 69% boost in citations over five years.

ResearchGate appears to be more expansive in terms of what can be hosted on the site and can include, for example, datasets, project updates, patents, working papers, code, and negative results. Users are encouraged to fill out their complete profile, and list awards and their previous work experience at a minimum. It also provides a forum for technical questions and answers, and most recently, allows users to group publications and data into ‘projects’. SE’s profile includes 418 contributions as of 19 ^th^ March 2017 and provides a reach of ca. 15,000 researchers, who can learn about his publications, presentations and postings to the site. It should be noted he also barely uses this software, as he prefers to put his efforts elsewhere. Academia and ResearchGate, when used to develop a network of followers, both offer an excellent communication path for sharing activities and research outputs with the community using the sites. These sites require some considerable effort to respond to requests and engage with others if you are to fully maximize their value.

*It is important to note that any uploads of published work to these services requires permission from the publisher.* A strong movement from publishers, which is beginning to take momentum, puts pressure on these services to ensure they educate their users on appropriate copyright. You may find the publisher will contact you if they feel that your published work is listed on ResearchGate and conflicts against your agreed copyright. To ensure that ResearchGate is used without risking a breach of copyright, it is necessary to read the Copyright Transfer Agreement or transfer license associated with the publisher to see how the paper can be shared. Open access policies can be checked on the journal’s website or by using SHERPA RoMEO, a site that presents policies based on a journal title or ISSN search.

### Sharing

Currently, the most widely adopted social sharing platform is Facebook, approaching two billion users as of May 2017. While we acknowledge the penetration, versatility and general global acceptance of Facebook as a platform, we collectively use it for sharing with friends and family mainly, only rarely sharing links to some of our scientific works. We prefer to separate career activities from personal pursuits, but acknowledge that this is also a personal choice. For younger generations this may be inverted, their followers on Facebook may be colleagues rather than family and for them it would be seen as appropriate to share on Facebook, on Instagram or on group chat site, such as WhatsApp. Instead, for sharing we use alternative online tools as outlined below.

***Blogs*.** All three of us have managed blogs for a number of years and two invested considerable amounts of time in sharing opinions, data and ideas ( SE and AJW). We have developed collaborations of value, asked for opinions and guidance and used them to share information regarding our (or others) latest research efforts, industry research and news/updates. Overall though, it seems that there is less interest in blogging, probably due to the amount of time needed to develop content and we ourselves more commonly share information now in smaller soundbites (via Twitter and LinkedIn). We also try to reach other networks by being guest bloggers on the blogs of others or news services and using these other sites to share data. There are so many other applications now available for sharing relative to just a few years ago that we will therefore focus our discussion on those sites that we use on a more regular basis.

***“Small nugget sharing”: Twitter/Google Plus*.** In many ways both Twitter and Google Plus are for sharing bite-sized communications, presently limited to only 140 characters for Twitter, but possibly to be extended. In terms of sharing, it is an ideal application for pointing your network to papers, publications, data sets, and blog posts via embedded short links. Networking retweets can drive attention to these various types of resources. It is simple to use and push out a URL to something you wish to share can drive attention easily, though only for a fairly short period of time. Once an item of interest has been prepared on some other form of social media, or a website link to a publication requires sharing, Twitter and Google Plus then become ideal ways to point to these items. Examples of nugget sharing include letting people know in advance that you will be at a conference (and potentially set up a “tweetup” with new connections), or live tweeting at a conference ^11^. One of the primary reasons that we use these platforms is for amplification of our publications ( *vide infra*). Both AJW and SE have initiated collaborations and specific data sharing activities via Twitter. For example, SE attended a meeting and shared the need for an application to build on a Green Chemistry dataset that was available only as a PDF file. What was initiated as a public Twitter exchange resulted in a mobile application being available within a few days ^12^, an example of a specific collaboration initiated via 140 character exchanges. Another example is a Twitter exchange that gained support for a new chemical identifier associated with the EPA CompTox Chemistry Dashboard added to Wikidata. Also, an open research project on Ebola (SE) was initiated, which in the space of two years facilitated the identification of new antivirals, publications ^13– 17^ and an eventual NIH grant. Others have described how Twitter can be used by scientists to extend network networks and ultimately find jobs ^18^ and, in our domain of chemistry, for sharing “ Real-time Chemistry”.

 Taking advantage of the communities of followers on Twitter and Google Plus is only possible, of course, once you have established a community. Building a community requires engaging in the platform by following other users who post interesting content, by engaging with the content, sharing other people’s content and posting your own. Developing your own following is an incremental process, which may take years and there are more expansive guidelines available on how to do this.

**Figure 3.**
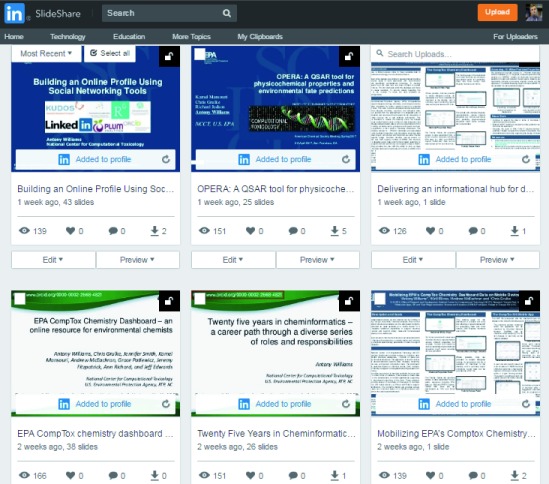
Powerpoint presentations associated with the SlideShare account of Antony Williams. SlideShare is part of the LinkedIn application family and one click is required to share the presentations to the LinkedIn profile and share them with all account followers.

***Media sharing: Presentations, preprints and videos*.** Most scientists present their work at conferences, either as talks or posters. Without using online sharing tools, the only people who would see your presentations, which commonly take hours of time to put together, would be limited to the people at the meeting. However, online tools for sharing and distributing these same presentations can result in a much broader reach, and importantly, keep the work alive for a period much longer than the limited presentation time, and associated audience, at the conference. One commonly used presentation sharing platform is SlideShare (acquired by LinkedIn in 2012). An advantage of these websites is that they both belong to the same company (Microsoft). This allows a relatively simple process for associating a presentation with your profile with one click (“Add to Profile”) to make the presentation visible ( Figure 3). SlideShare is not limited to simply sharing PowerPoint presentations: the user can include article preprints, infographics and other documents, as well as the ability to have integrated videos embedded via YouTube. One approach (adopted by AJW) to derive most value from the network effect of multiple connected platforms, reach the broadest audience, and share the work in various forms is as follows: 1) a PowerPoint presentation delivered at a meeting is shared on SlideShare (and also figshare, ResearchGate and Academia); 2) a narrated version with voiceover to capture the presentation is made and published to YouTube; 3) the YouTube embed function is used to insert the video to the second slide on SlideShare; and, 4) a viewer will then have the choice to view the slide deck, download it for local storage, and if they want they can hear the author also present the slide deck with the voiceover.

While we acknowledge that the most popular global video sharing platform is YouTube, there are geographical issues, not only based on language, in terms of all countries accepting the sharing platform. Streaming content in China via YouTube is an issue and other platforms, such as Vimeo or Weibo, may be an option. We think that scientists should not mix their scientific movies (for example, narrated presentations, lab activities, etc.) with family movies!

**Figure 4.**
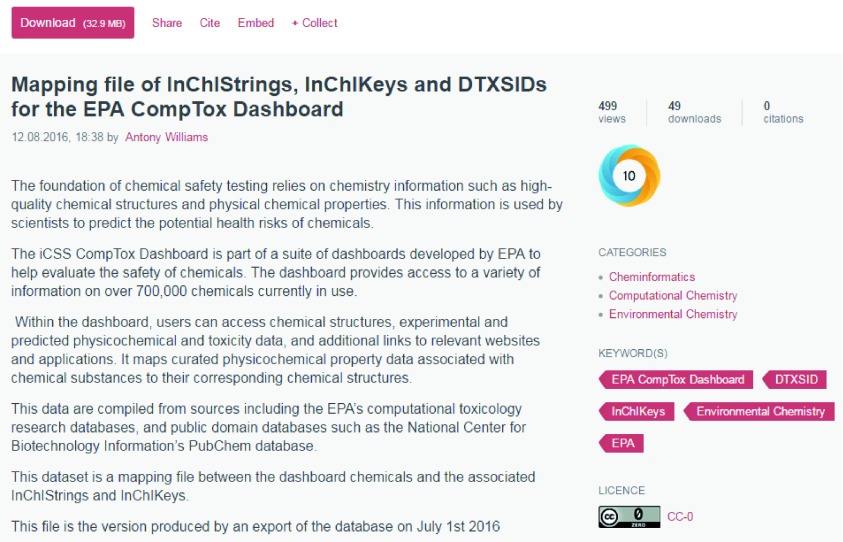
A published dataset on figshare showing the number of views, downloads and citations, which also includes an associated “Altmetric donut”. At deposition of the file, a digital object identifier (doi) can be requested which, for this file, is accessible at https://doi.org/10.6084/m9.figshare.3578313.v1.

***Data sharing*.** There are myriad platforms available for data sharing, and it is difficult to be exhaustive in this short article as, depending on the particular domain of science, there will be biases. Climate science, versus chemistry, physics or medical sciences have their own favorite platforms. We have experience of the Dryad Digital Repository, figshare and Mendeley data for sharing most data types. Other sites can be used for specific types of data sharing. For example, PubChem for sharing BioAssay data. For the purposes of this article we focus only on the figshare site for data sharing, as we have the greatest experience in terms of using this platform, as well as the fact that it is now integrated with many publishers that we have published our articles with (i.e. PLOS, the American Chemical Society, Springer and Wiley). Importantly, figshare offers the advantage of creating DOIs that give unique persistent identifiers that can then be resolved across platforms. Datasets published to figshare can be embargoed, cited in a manuscript, and made open at the time of publication. This provides important benefits as now specific datasets are fully citable (via DOI), the number of views and downloads are directly tracked, the altmetrics can be measured and, overall, there is significantly more insight into how data is used and accessed over simply putting a file as supplementary info with a publication. An example of a shared dataset, including views, downloads and an associated “Altmetric donut” is shown in Figure 4. The donut represents the Altmetric Attention Score and is designed to identify how much and what type of attention a research output has received. The colors of the donut represent the different sources of attention and the details regarding how the score is calculated are available online.

With the data (or presentations or documents) on figshare, we can then share details via Twitter and use the associated DOI to cite our datasets again on ResearchGate. figshare also allows us to share figures before they are used in manuscripts, define them with a CC-BY Creative Commons license, and then use them in our publications. In this way, we are not transferring copyright of our figures to the journals and other people who wish to use our figures do not have to request permission as they are already in the public domain. figshare is also a platform where we share our presentations, posters and preprints (other preprint servers do exist, such as arXiv, bioRxiv, and the impending ChemRXiv. It is also where we tend to host our data that we point to from our publications, as well as using subsets of the overall data in the supplementary information with publications. Tagging of any form of information that we share via the site makes it more discoverable, and a search across a specific application website can therefore surface this to interested parties. More recently figshare has added ‘collections’, and we have made use of this around our work on the Zika virus. As multiple academic publishers have now accepted figshare as a data repository of choice, its reach appears to be growing and it is likely to have increasing importance as a data repository for science.

***Code sharing*.** It is advantageous to those that produce computer code, as a part of their scientific output, to be able to share it and allow others to consume it. Major code repositories, such as GitHub and SourceForge, offer many advantages for collaborative software development and versioning, so have great utility in their own right. They are integrated into online sharing platforms, thereby ensuring that code updates are transmitted across the community, keeping the audiences, who may require code access, informed of new depositions that they can consume. Most scientists may never use these repositories, but they are of central importance in the science ecosystem and other data sharing tools can learn from them. There are other reviews regarding the adoption of these platforms and readers are pointed to these for more detail ^19^. We, like many others, believe that code used in projects that is then used to deliver software underpinning, for example, data processing, analysis and reporting, should be citable and ultimately be a part of the altmetrics feeding a scientist’s acknowledgement for their contributions to science.

### Impact tracking

As scientists, one of our interests is to track our publication records, have access to our citation statistics, and, potentially measure the impact of our work. Impact can be estimated by a number of statistics, such as the h-index. There are even programs available so you can generate your own statistics.

In recent years, catalyzed specifically by the work of Priem *et al* and the release of the “ altmetrics manifesto”, altmetric statistics have started to gain general acceptance within the community as measures of interest in a scientist’s work. This acceptance is likely to increase as their algorithm becomes more mature and produces increasingly relevant results. Altmetric statistics not only take account of standard publication metrics, such as citations, article views and downloads, may also track views and downloads for presentations and videos, and include measures of attention for discussion (via blogs) regarding publications online on platforms such as Twitter and Facebook. Altmetric statistics may also attempt to measure the impact of the reuse of data sets and code. This, to many, may seem incredibly complex and go far beyond just tracking “the paper” itself, but this is the world that is evolving. A number of tools attempting to integrate and track these altmetrics impact statistics have been established. These include ImpactStory, Altmetric and PlumX. This section discusses some of the sites we use for managing our publication records and tracking our classical citation statistics, as well as those that we use for measuring our impact in the realm of altmetrics.

For the purposes of “publication and citation tracking” we use: ORCID, Google Scholar, and Microsoft Academic Search. While other platforms, such as ResearchGate, do an excellent job of informing us via emails when there are new publications to associate with our profile, once we have confirmed the associated publication as appropriate, we use it more as a networking site and catch-all for the bulk of our research outputs (see earlier). These three sites are more focused on simply tracking publications and, in the case of ORCID, it expands to include presentations listed on figshare (via the DOI integration). Each of these websites is free to use for an individual scientist, while ORCID also offers access to an institutional package ( *vide infra*) that allows organizations to mesh together contributions for their staff into an institutional representation of activity.

***ORCID*.** An Open Researcher and Contributor Identifier (ORCID iD) is a unique numeric identifier for a researcher that is free to claim and can be obtained simply by registering at the website. Almost four million identifiers have been claimed (or issued) at the time of writing. The ORCID iD is a derivative of previous efforts by Clarivate Analytics (formally Intellectual Property and Science business of Thomson Reuters) to produce the ResearcherID that has a distinct benefit of disambiguating authors and their association with publications, which is an issue for other sites (see later for a distinct example of this problem with Google Scholar). Once a scientist has claimed their unique identifier, they are responsible for defining the content associated with it, including whether they wish the content to be public or private. They can add a short biography and associate a number of their websites and public profiles with the ORCID site. Increasingly, these identifiers are expected or accepted by publishers at the time of manuscript submission, and funding agencies are also starting to use them. The website allows for an online resume to be assembled from publications by searching based on your name and editing the list as necessary. The data collected are then available via an application programming interface and, for example, can be used by publishers to use on their own platforms for enhancing the linkages between an authors’ publications. As a starting point, it is possible to upload a list of publications in a standard format, such as Bibtex or EndNote. It is also possible for a scientist with a ResearcherID to connect and migrate the existing content to ORCID and expand from that point. The ORCID application programming interface and authorization module allows connectivity between web-applications.

 Since the ORCID iD itself has value independent of the capabilities of the website as a representation of a scientist’s publication record and resume, obtaining an ORCID iD is, in our estimation, one of the primary entry points into the scientific networking regime today and we encourage registration. Inclusion of the ORCID into PowerPoint templates for presentations shared online, and on other scientific networking and data sharing sites ensures that a simple web search in the future will aggregate the majority of your public works labeled with the identifier. We also add the ORCID iD either on the first or last slide of our slide decks with the intention that it is captured by the search engines, allowing a simple search to provide us a list of ORCID indexed works.

***Google Scholar and Microsoft Academic Search*.** Google Scholar (GS) is a free website for assembling what is effectively a list of your publications and their citations. GS also provides metrics, such as the h-index, that we have found to be generally ** much ** higher than in the Web of Science (WoS), which could be because it includes self-citations, whereas WoS does not. There have been comments that GS is a better predictor of actual citation numbers than WoS. GS is useful for searching for publications and perhaps picking up citations that the commercial tools are missing. We have seen a worrying recent trend in terms of auto-associating publications, and recently AJW identified that 70 publications had been associated with his GS profile and had to be manually deleted. Profile maintenance is therefore necessary by the user, and there has to be careful curation and pruning on an ongoing basis. Microsoft Academic is much like GS, but we have found it to be less useful because it was unable to capture citations to our publications.

***“Alternative metrics”: Plum Analytics, Altmetric, ImpactStory*.** These tools aggregate the citations from blogs, tweets, Facebook, etc., and use their own algorithms to derive a score for each paper. Twitter is commonly a major contributor in terms of social media counts, and a weighted approach in terms of the importance of a social media event can be taken into account. For example, Altmetric give a news item a higher weighting than a tweet. Interestingly, ResearchGate also derives a score for each author, though it remains a little unclear how the score is calculated.

### Amplification

**Figure 5.**
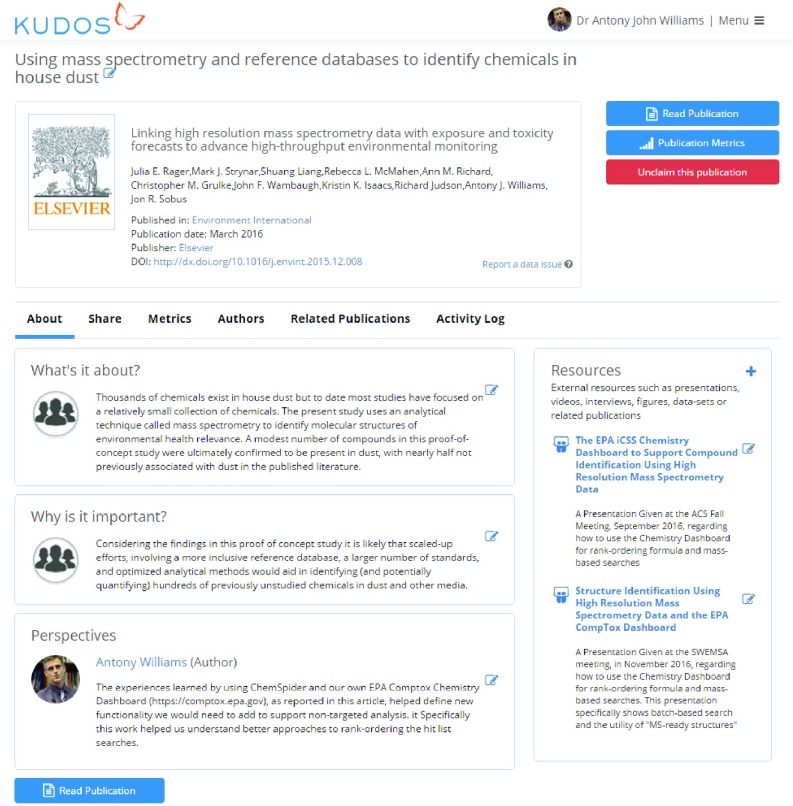
Kudos tracks citations, alternative metrics (e.g. Altmetric), and other statistics, such as downloads and clickthroughs. External resources such as presentations, videos, interviews, figures, datasets or related publications can be linked to an article as is shown (right hand side). The claimed article is available directly by appending the DOI to the growkudos.com URL ( https://www.growkudos.com/publications/10.1016%252Fj.envint.2015.12.008).

***Kudos*.** The emerging area of author support tools has very limited research findings available (e.g. data and code). However, Kudos has been recently highlighted as useful in this regard ^20^. While there are plenty of websites for an author to post their papers and preprints, enriching these research outputs to add more information about what commentaries have been made about the work, linking to additional presentations, datasets, etc., is less supported in general. Kudos tracks citations (supported only by WoS statistics at present), altmetrics on publications (supported by Altmetric currently), and other statistics, like usage, where available (e.g., downloads and clickthroughs). It also provides a dashboard of an author’s papers using a CrossRef DOI as the basis of the data feed and can be linked to your ORCID account ( Figure 5). For a new user there is a downside if they have a large publication record, as it will take a very long time to enrich every publication with links to other content, but a user can of course choose to ignore their historical record of publications and focus only on new publications moving forward. Our adoption of the platform has allowed us to enrich publications with information (with examples described below) and share the associated Kudos page, drive traffic to the paper and track this activity. In our experience, Kudos results can be improved when multiple authors contribute and work together to improve awareness of a publication because the publication is disseminated through multiple-author networks and online networking efforts.

 The enrichment capability offered by the Kudos platform delivers a valuable capability to the authors of the publication: the ability to keep the publication up to date by linking to related information, for example, blog posts regarding the work, media coverage, presentation slide decks from conferences, later publications by the authors themselves or derivative works by other scientists. One of the authors (AJW) coined the term “forward citation” to refer to this capability, as citations are retrospective in nature and point only to earlier work. Enrichment of an article can continue to keep the research reported in a publication updated with follow-on information as later works of various types are associated. An example of this from the author’s list of publications is associated with the synthesis of the chemical known as “Olympicene”, a small molecule synthesized during the time of the 2012 Olympic Games as a form of molecular commemoration. The claimed publication has been enriched with a YouTube movie, multiple blog posts, various types of media coverage and even detailed discussions regarding part of the chemical synthesis. In particular, multiple scientific papers by other authors referring specifically to the trivial chemical name of Olympicene have been associated with the original paper thereby connecting the work directly. While search engines and referential systems from publishers attempt to do this in an automated manner, in this case the claiming author(s) have an opportunity to directly make the association and comment as appropriate, regarding the value and nature of the related information.

In June 2016, Kudos and the Altmetrics Research Team at the Centre for HEalthy and Sustainable CitieS (CHESS), and the Wee Kim Wee School of Communication and Information at Nanyang Technological University (NTU, Singapore) analyzed user data and found that 51% of registered Kudos users were STEM researchers sharing their work, 29% were social sciences researchers and 8% were humanities researchers. This demonstrates the engagement of STEM researchers, and how they are leading the way in using innovative tools to disseminate their research. However, with seven to nine million active researchers a year ^3^, Kudos only has around 1% penetration into the research community, so there is huge growth potential here for these types of services to help more of the scientific community.

### Organizational impact

This article has primarily focused on the benefits of these tools for the individual researcher in terms of sharing their research and work outputs in the forms of data, presentations, publications and other outputs. A number of the tools also offer capabilities to track “organizational impact” ( Kudos; Altmetrics; ORCID). LinkedIn already allows aggregation of users into organizations so that they can stay informed, connected and follow and post updates (see here and here). Being aware of your institution’s research in terms of what is being disseminated, what the comments are out in the public domain via the social networks and media is certainly of interest to any organization. For example, Altmetrics data can help track the influence of an institutions work on public policy and helps provide insight into value of research outputs. Since these tools can give almost immediate details about the public engagement with work outputs as soon as it is published, this ensures that it is bring interpreted correctly and addresses any potential issues resulting from follow on reports. In terms of funding, many of these sites can be valuable in engaging new collaborators and sponsors, as well as providing information valuable in obtaining future support. Online activity benchmarking relative to other peer organizations can be important in terms of identifying contributions and productivity.

## Potential downsides to this new alchemy

This article has outlined various tools and approaches than can be executed in order to develop an online network and has defined a number of benefits resulting from participation. For balance, it is appropriate to also identify potential downsides to engaging in these activities. It is certainly true that there is a potential for noise in the network as millions of people have moved online and share their views, commentaries and concerns. In the domain of science, as secluded as it may be from the engagement of the masses, if all scientists were to take advantage of the software applications to share their activities, the advantages endowed upon the early participants in online networking for scientists could be at risk to increase the noise in the system. Separating the true signals from the noise will require development of necessary skills to participate online in a manner that rises above the noise. There is likely more to lose by not participating, than by working to develop an online presence that both contributes to the greater community and develops your own following.

Online platforms will certainly be used to push both good and bad, or weak, science. However, these platforms also offer opportunities for true scientific discourse that will assist those who have less scientific knowledge to other sources of information, with the potential to re-educate and advise. While bad science has already developed a voice online, there is an active community just as willing to participate in the debate and reactions can be swift.

 Science is meant to be data-driven and objective, yet open to discussion and reinterpretation relative to multiple hypotheses. Historically this has been paced relative to the release of relevant publications to the public. Peer-review has been limited to a small audience prior to publication, commonly between three and five scientists, and then released to a larger community for consumption. Historically, responses to articles would be based on letters to the editor and would be slow to move into press, likely with exchanges behind the scenes between editors, authors and potential critics. Post-publication peer-review is now facilitated by publishers, allowing direct comments to be posted against articles, in general with moderation, but *any* publication can now be critiqued immediately after release. Naysayers to scientific work can be summarily disregarded and their commentaries debated in the public domain. Meanwhile online networking tools also provide an exciting and engaging means to cautiously discuss science, and even conduct further work in the laboratory to validate the reported findings. From our own domain, the Hexacyclinol controversy was taken online to a discussion with a community of interested scientists, and while the community disassembled the science published in February 2006 it was a full six years before retraction ^21^, this after dozens of blog posts and online discussions. Similarly, an article regarding *oxidation* by a reducing agent ^22^, sodium hydride, was dismissed by “ peer review in the blogosphere” in a matter of days following publication. This included blog posts from labs showing NMR spectra and detailed exchanges between scientists on blogs. Sadly *all* of this discourse failed to make it to the journal article where the simple message communicated by the American Chemical Society on the journal page is “This manuscript has been withdrawn for scientific reasons” ^22^, and the science reported online has been lost to posterity as a result of the majority of links decaying into obsolescence (i.e. http://www.coronene.com/blog/?p=842 and http://www.organic-chemistry.org/totalsynthesis/?p=1903; both fail to link to the original posts). This points to the somewhat temporary nature of internet exchanges and the challenge of maintaining and archiving these for future retrieval.

 Hundreds of millions of tweets are exchanged every day, an average of 6000 per second as of this writing. Blog posts are loaded and commented on. The number of English Wikipedia pages is approaching 5.5 million, with about 750 pages added every day, and multiple edits being made at any point in time. Can we depend on the Internet Archive Wayback Machine to capture all of this content? While the Wayback Machine *did* capture the decayed page regarding the oxidation by sodium hydride ( https://web.archive.org/web/20090801231430; http:/www.coronene.com/blog/?p=842), it is highly unlikely that capturing all internet knowledge is even feasible as the machine takes irregular snapshots. As with the information contained within books, as with knowledge itself, internet content can decay and morph yet society, science and humanity continues to move forward unabated. Not all contributions and engagements in the online networking world will make a difference and we can only hope that there is useful signal in the noise.

## Conclusions

The public online networking, tracking and amplification tools described in this article that can be used for raising awareness of scientific outputs are just the tip of the iceberg. We acknowledge that our efforts invested in them would dissipate if the software tools cease or are overtaken with new offerings. For example, WhatsApp, with a worldwide user base of 1.3 billion users, is not used by any of the authors yet! We are not alone in terms of ignorance of the app; however, it was also omitted from the recent Times Higher Education listing of social media for academia. Perhaps this app represents an untapped tool for communication and networking in science. We imagine that the software tools used in five years by scientists are likely to be different, though some of the existing sites will persist, so there may be a hurdle to overcome before engaging with a new software application. Social media tools overall can be viewed as a conversation container that is most relevant when it is current and with value that degrades over time; i.e. a tweet from five years ago might not be as relevant as when it happened. This also raises the question of longevity, as these efforts would not be around as long as the papers, which could be useful for many years or decades.

Adopting a new application must either offer some specific advantages over existing apps or include ways to incorporate your information to avoid re-entering data. If you want to learn more about our personal use of online media for science communication and how it has evolved over the years, please review our supplemental materials ( https://www.slideshare.net/AntonyWilliams/; https://www.slideshare.net/ekinssean/). Our involvement in sharing data, research activities, presentations and publications has developed over a period of almost a decade. AJW has presented dozens of times at educational institutions and governmental organizations. SE most recently presented his experiences at the AAPS conference. LP has supported hundreds of scientists and authors in numerous disciplines over the years to help them publish, disseminate and increase the impact of their research. In addition, LP also advises publishers and libraries on how best to support their researchers. During these efforts, there are a couple of common observations. Adoption of online media tools appears to be generational with much faster uptake by early career scientists and later generation scientists generally avoid them. Some scientists see participation in the use of open data-sharing, posting their presentations and putting efforts into amplifying their research as not an appropriate or useful activity. Similarly, there appears to be true advocates of openness, especially with the increasing drive towards open access publishing, but we have certainly met scientists who take a very neutral or skeptical position on open science and sharing in general.

One of the recurring themes of our engagements is that many of these software tools exist in isolation and there is no way to link them all together, thereby requiring multiple efforts to populate them with data and information. This results in repetitive efforts and time wasting by users. It does, however, present a potential commercial opportunity to support those with little time to invest in starting or maintaining use of these various tools, which could enhance the visibility of their scientific outputs. A useful service would be to offer an integration tool to update multiple online media profiles, with at the very least the most basic of information, and to show the relative benefits from these different tools. Or one could set this as a project for their younger lab members who might be more adept with the technologies!

While we have mentioned a number of online platforms that we use in our own efforts to network, share and amplify our research, these are not necessarily the best tools available for every individual scientist’s use case. AJW primarily operates in the field of chemistry, cheminformatics and chemical-biology, while SE is focused more on drug discovery for rare and neglected diseases, and our chosen tools are based primarily on our early adoption, familiarity and cross-fertilization from collaborating over the past decade. The most appropriate sites for physicists and biologists to share their data may well be very different. There will be more websites and applications coming online in the future that may be even more fit-for-purpose for a scientist operating in a particular field. We encourage experimentation and adoption, if you find them of benefit. To begin with we suggest keeping it simple, use a few tools, and focus on fundamentals – be smart with the time you have available. We find ORCID identifiers to be increasingly in demand by publishers and they will be an expected part of every scientists’ profile before long. Our Google Scholar Citations profile are our primary method by which to track publications and, as a beneficial side effect, inform us of citations to our work. LinkedIn is the primary professional networking site at present and it is worth the effort to develop an extensive profile. SlideShare (or similar) is valuable for sharing presentations and documents, figshare (or alternatives) for sharing citable data, Kudos for post-publication enhancement by associating with later or relevant works, and Twitter for bite-sized sharing into a large network of potential engagement. While a scientist may not see much traction with one tool, coordinating how to use more than one is the key, which should lead to seeing the benefits from engaging with this ‘new alchemy’. We think you will quickly discover what works best by measuring activities and what gives the most impact, as it may be different for each scientist.

The approaches outlined here regarding sharing details about a scientific manuscript, or simply a research study and associated data, also offer a number of potential positive effects that can contribute to the quality of science. The historical approach of peer review was to, hopefully, both improve and ensure the quality of the science and the published output. Sharing research data, presentations, posters and preprints allows for early feedback on both the results and preliminary findings and thereby offers the opportunity to get feedback from peer groups and reviewers. This can certainly help contribute to the quality of science before the final published record in a journal is established.

## Disclaimer

The views expressed in this article are those of the authors and do not necessarily reflect the views or policies of the U.S. Environmental Protection Agency. Mention of or referral to commercial products or services, and/or links to non-EPA sites does not imply official EPA endorsement.

## Author information

Twitter accounts for AJW, LP and SE can be viewed at:

AJW: @ChemConnector

LP: @loupeckconsult

SE: @collabchem

## References

[1] Collins K Shiffman D Rock J: How Are Scientists Using Social Media in the Workplace? *PLoS One.* 2016;11(10):e0162680. 10.1371/journal.pone.0162680 27732598 PMC5061391

[2] Jinha AE: Article 50 million: an estimate of the number of scholarly articles in existence. *Learn Publ.* 2010;23(3):258–263. 10.1087/20100308

[3] Ware M Mabe M: The STM Report.2015 Reference Source

[4] Willams AJ Pence HE: The future of chemical information is now. *Chem International.* 2017;39(3), In press. 10.1515/ci-2017-0304

[5] Bohannon J: Who's afraid of peer review? *Science.* 2013;342(6154):60–5. 10.1126/science.342.6154.60 24092725

[6] Sorokowski P Kulczycki E Sorokowska A: Predatory journals recruit fake editor. *Nature.* 2017;543(7646):481–483. 10.1038/543481a 28332542

[7] Van Noorden R: Controversial impact factor gets a heavyweight rival. *Nature.* 2016;540(7633):325–326. 10.1038/nature.2016.21131 27974784

[8] McEachran AD Sobus JR Willams AJ: Identifying known unknowns using the US EPA’s CompTox Chemistry Dashboard. *Anal Bioanal Chem.* 2017;409(7):1729–1735. 10.1007/s00216-016-0139-z 27987027

[9] Jamerson J: Microsoft Closes Acquisition of LinkedIn.In *Wall Street Journal.* 2016 Reference Source

[10] Niyazov Y Vogel C Price R: Open Access Meets Discoverability: Citations to Articles Posted to Academia.edu. *PLoS One.* 2016;11(2):e0148257. 10.1371/journal.pone.0148257 26886730 PMC4757559

[11] Ekins S Perlstein EO: Ten simple rules of live tweeting at scientific conferences. *PLoS Comput Biol.* 2014;10(8):e1003789. 10.1371/journal.pcbi.1003789 25144683 PMC4140634

[12] Ekins S Clark AM Williams AJ: Incorporating Green Chemistry Concepts into Mobile Chemistry Applications and Their Potential Uses. *ACS Sustain Chem Eng.* 2013;1(1):8–13. 10.1021/sc3000509

[13] Ekins S Freundlich JS Coffee M: A common feature pharmacophore for FDA-approved drugs inhibiting the Ebola virus [version 1; referees: 2 approved]. *F1000Res.* 2014;3:277. 10.12688/f1000research.5741.2 25653841 PMC4304229

[14] Ekins S Coffee M: FDA approved drugs as potential Ebola treatments [version 1; referees: 1 approved, 1 approved with reservations]. *F1000Res.* 2015;4:48. 10.12688/f1000research.6164.2 25789163 PMC4358410

[15] Ekins S Southan C Coffee M: Finding small molecules for the ‘next Ebola’ [version 2; referees: 2 approved]. *F1000Res.* 2015;4:58. 10.12688/f1000research.6181.2 25949804 PMC4406187

[16] Ekins S Freundlich JS Clark AM: Machine learning models identify molecules active against the Ebola virus *in vitro* [version 2; referees: 2 approved]. *F1000Res.* 2015;4:1091. 10.12688/f1000research.7217.2 26834994 PMC4706063

[17] Litterman N Lipinski C Ekins S: Small molecules with antiviral activity against the Ebola virus [version 1; referees: 2 approved]. *F1000Res.* 2015;4:38. 10.12688/f1000research.6120.1 25713700 PMC4335594

[18] Baker M: Social media: A network boost. *Nature.* 2015;518(7538):263–5. 10.1038/nj7538-263a 25679032

[19] Thung F Bissyande TF Lo D: Network Structure of Social Coding in GitHub. In *Software Maintenance and Reengineering (CSMR)*, 2013 17th European Conference on. *IEEE.* 2013 10.1109/CSMR.2013.41

[20] Perkel JM: Scientific writing: the online cooperative. *Nature.* 2014;514(7520):127–8. 10.1038/514127a 25279924

[21] La Clair JJ: Retraction: Total syntheses of hexacyclinol, 5- *epi*-hexacyclinol, and desoxohexacyclinol unveil an antimalarial prodrug motif. *Angew Chem Int Ed Engl.* 2012;51(47):11661. 10.1002/anie.201206869 23297422

[22] Wang X Zhang B Wang DZ: Reductive and transition-metal-free: oxidation of secondary alcohols by sodium hydride. *J Am Chem Soc.* 2011;133(13):5160. 10.1021/ja904224y 19621929

