# Supplementary material for: The new alchemy: Online networking, data sharing and research activity distribution tools for scientists
Source: F1000Res. 2017 Aug 3;6:1315. [Version 1] doi: 10.12688/f1000research.12185.1 (PMC5580431; doi:10.12688/f1000research.12185.1)
Supplement: Peer review history

# Peer review: Referee response for version 1

**DOI:** 10.5256/f1000research.13189.r24790

**Published:** 2017-08-24

**Authors:** Matthew R. Hartings

**Affiliations:** 1 Department of Chemistry, American University, Washington, Dc, USA

The expanding world of on-line dissemination and sharing and conversations surrounding scientific research can be daunting for many scientists. Williams, Peck, and Elkins are certainly qualified and experienced in this area. And, their article "The new alchemy" will certainly be useful to many as they try to figure out how best to navigate this online environment. 

 The greatest benefit to this article, is the clear description of what the different on-line platforms are meant to be and what they are best used for. Readers are sure to find the authors examples and guidance helpful. I am glad that they have written this manuscript and think that F1000 is an ideal place for publication. 

 There are two issues that I wish the authors had commented on a little more extensively. 

 The first is "impact." I appreciate that the article is meant to lay bare what tools are out there for scientists to use in sharing their research. I also appreciate that "impact" is a loaded term and is exceedingly difficult to gauge (and is often done poorly). But, it remains that it is what many scientists focus on. I have personally found that tools like Plum Analytics and ImpactStory and Altmetric are useful in helping me to compare my article-level metrics to those of my peers and to those of other articles published in the same journal. What these tools specifically try to measure is the effectiveness of how well you are able to share your content across these online tools that the authors describe. Ultimately, we scientists need a reason to share our work. For selfish reasons, I think that many scientists will find benefit to these "impact" tools. 

 The second theme that I would have liked the authors to frame slightly differently is "post-publication peer review." The scientists who engage with F1000 do not need to be told the benefits of post-publication peer review. However, it would have been gratifying for me to see the authors list sites that have post-publication review options as a way of sharing research rather than just in the sections on "potential downsides." Science is not static. That sentiment goes for published science as well. Scientists would be wise to welcome and actively engage with post-publication peer review. It is this active engagement that is key for making sharing worth-while. Sharing for the sake of sharing is only a half-step. If a scientist wants to have lasting effects, fully engaging in the process of sharing is absolutely required. 

 I applaud the authors for putting this article together. I know that there are many within the scientific community who can benefit from their commentary.

I have read this submission. I believe that I have an appropriate level of expertise to confirm that it is of an acceptable scientific standard.

# Peer review: Referee response for version 1

**DOI:** 10.5256/f1000research.13189.r24792

**Published:** 2017-08-18

**Authors:** Alice Meadows

**Affiliations:** 1 ORCID Inc., Brookline, MA, USA

This is an interesting paper that is hard to classify, as it doesn't fit neatly into any one category of article. It is a mix of best practice recommendations for and a review of online networking, data sharing, and research activity distribution tools for scientists - a blend of fact and opinion, part editorial and part research paper.

 On the plus side, this is a very helpful overview and evaluation of many of the ever-increasing tools and services available to help scientists promote and share their research online, from a group of people who understand why and how to use them. Gathering all this information into a single paper is a real service to the community and should, I hope, prompt more scientists to follow the authors' advice, and : "encourage further exploration to find out what tools work best for you to meet your objectives, and even form the foundation to discover new or other tools not identified in this piece."

 However, the paper is weakened by the fact that there are a number of points when the authors express their own opinion without providing any evidence to back it up. For example, from the Introduction: "We believe there are a number of reasons that most scientists do not use these tools. It could be because few people would even think of using online media tools for their scientific research, or because they do not understand the potential value. The lack of credit for sharing pre-published data, code or other forms of research outputs, especially in terms of citations that can contribute to career progression, may also be an issue. Maybe scientists do not see this activity as a valuable use of their time or they require initial guidance to help navigate use of these tools.” Or, from Categories of Tools (Networking): "While LinkedIn has a primary role to form connections and expose your career to potential employers and partners, it is likely the *de facto* networking tool for many professional scientists." These and other unsupported statements are most likely correct, however, they would be more credible if supported by evidence in the form of published research, as is the case for much of the paper.

 By the authors' own acknowledgement, the article is based largely on their own experiences. They are clearly expert and long-term users and consumers of the tools and services they describe, which makes the information they share very helpful, especially for those who are less expert. But their experience is also, inevitably, limited by their own geography (USA and UK) and specialisms (chemistry). So the challenges of sharing via social media in China, for example, only get a brief mention. And as they point out: "these are not necessarily the best tools available for every individual scientist’s use case... The most appropriate sites for physicists and biologists to share their data may well be very different."

 Overall, I found this a very thorough and helpful contribution to the literature on online tools and services for researchers. It would be even more valuable if could be updated regularly with information about new tools and services, taking into account the needs of a wider population of researchers by discipline and region.

I have read this submission. I believe that I have an appropriate level of expertise to confirm that it is of an acceptable scientific standard.
